# Comparison of Stock and Customized Implant Abutments Regarding Peri-Implant Health: Comparison of Stock and Customized Implant Abutments

**DOI:** 10.31661/gmj.v13iSP1.3595

**Published:** 2024-12-31

**Authors:** Hanieh Ehtesham, Seyed Mohammadreza Hakimaneh, Maryam Tehranchi, Sahel Shahrestani

**Affiliations:** ^1^ Shahed University Faculty of Dentistry, Tehran, Iran; ^2^ Prosthodontics Department, Shahed University Faculty of Medical Sciences, Tehran, Iran; ^3^ Periodontology Department, Shahed University Faculty of Dentistry, Tehran, Iran

**Keywords:** Dental Implants, Dental Implant-Abutment Design, eri-Implantitis, Periodontal Index

## Abstract

**Background:**

Dental implants are one of the most predictable treatment options for replacement of the lost teeth. One of the factors affecting the success of an implant, is the health of the periodontium around the implant abutment. Recently, various materials have been used for fabrication of implant abutments. This study aimed to assess early marginal bone loss (MBL) and periodontal parameters around dental implants with titanium stock abutments and customized abutments fabricated by the computer-aided design/computer-aided manufacturing (CAD/CAM) technology to compare their success rate during a 1-year period.

**Materials and Methods:**

This prospective cohort study was conducted on 64 patients whose treatment plan included the stock abutment, and CAD/CAM customized abutment that were randomly selected. All patients underwent a clinical periodontal examination on the day of prosthetic crown delivery, and parallel periapical radiographs were obtained. The probing pocket depth (PPD), papilla bleeding index (PBI), keratinized gingiva width (KGW), plaque index (PI), and modified gingival index (MGI) around dental implants were calculated and recorded. Periodontal and radiographic indices, and MBL were measured at the one-year follow-up.

**Results:**

The stock and customized abutments had no significant difference regarding MBL, PPD, PBI, MGI, PI and KGW. The survival rate and success rate were equally 100% in the two groups.

**Conclusion:**

The two abutment types had no significant difference in any clinical parameter. Thus, stock or CAD/CAM customized abutments may be selected according to secondary parameters such as software availability, personal preferences, ease of fabrication and cost.

## Introduction

Dental implants are currently the most suitable option for replacement of the lost teeth due to their high survival and success rate [1]. However, despite the high survival rate of dental implants, complications such as marginal bone loss (MBL) are still likely to occur [2].

MBL is a multifactorial process that occurs at the cervical level of dental implants and it is a key factor in development of peri-implantitis [2].

surgical trauma, occlusal trauma, gingival biotype, abutment micromovements, frequency of opening and tightening of the abutment, bacterial colonization at the abutment-implant interface, distance between the implant-abutment junction and bone crest, and macro or micro-geometry of dental implants may play a role in the occurrence of MBL [3]. Furthermore, optimal implant-abutment connection plays a fundamental role in peri-implant hard and soft tissue stability [4].

The long-term success of implant abutments depends not only on selection of a suitable and biocompatible material, but also on the abutment design and fabrication process. The abutment design and geometry significantly affect stress distribution in implant-supported crowns. Also, considering the recent advances in digital technology and computer-aided design/computer-aided manufacturing (CAD/CAM) systems, it is important to evaluate the effect of the abutment design and fabrication process on the clinical outcome of the treatment [5].

To select a suitable abutment, dental clinicians should have sufficient information about the abutments and the influential determinants that affect this decision [6]. Stock abutments may not be used for all cases due to intra-oral condition or availability limitations related to the suppliers [7]. The shortcomings of stock abutments that are related to their difference with the natural tooth anatomy can compromise the proximal and buccal soft tissue support [7]. Resultantly, customized abutments gained increasing popularity due to reasons such as correction of implant angulation and gingival contour preservation [8]. They can also be fabricated by the CAD/CAM technology. The CAD/CAM technology controls the geometrical shape of the abutment, and enhances the adaptation of the external surface of the abutment with the adjacent natural teeth. It also contributes to a healthy gingival margin by minimizing the risk of residual cement accumulation in the gingival sulcus. Moreover, it controls the finish line of the abutment and prevents sharp borders in the abutment design. Accordingly, poor implant angulation may be compensated to some extent [7].

Furthermore, in order to use a standard abutment, dental implants should be in a relatively ideal position. The required conditions for using a standard abutment are limited, depending on the vertical position of the implant. A standard abutment cannot be used for prosthetic restoration of a deeply placed dental implant with screw-retained crowns because it cannot provide sufficient support for the ceramic crown. The abutment can be designed through wax-up so that it provides ideal support for the final screw-retained crown and excellent adaptation with the margin of the cement-retained crowns [9].

To the best of the authors’ knowledge extensive clinical studies are not available about the effect of the customized abutments on the periodontium, especially in Iran. Thus, this study aimed to assess early MBL and clinical health of peri-implant tissue around stock abutments and customized abutments fabricated by the CAD/CAM technology to compare the success rate of these two abutment types in a 1-year period of time.

## Materials and Methods

This prospective cohort study was carried out in order to compare MBL and periodontal indices around stock and customized abutments with ethics approval by the ethics committee of Shahed University: IR.SHAHED.REC.1400.050 on 64 adults presenting to a dental clinic in Tehran from March 2020 to August 2021 requiring implant restorations. The study population was divided into two groups; 28 stock abutments and 36 customized abutments using G-Power version 7.3.1.9 assuming α = 5%, study power (B-1) of 80%, and effect size of 71.0.

The inclusion criteria of this study were; (I) implants inserted in the posterior maxilla or mandible, and (II) healthy periodontium around implants at the session of prosthesis delivery, characterized by absence of bleeding, edema, gingival recession, peri-implant bone loss and availability of at least 2 mm keratinized tissue.

The exclusion criteria were; (I) severe bruxism, (II) history of untreated periodontal disease, (III) history of implant failure, (III) ASA score ≥ 3, (IV) history of head and neck radiotherapy, and (V) age under 18 years.

The patients with the inclusion criteria were then divided into two groups randomly. All patients received DIO implants (DIO Implant Co., Busan, Korea).26 implants were inserted in the maxilla and 38 implants were placed in the mandible.

. After implant placement surgery, radiographs were obtained and patients with properly inserted dental implants were enrolled. This was done to eliminate the confounding effect of surgery-related factors. Next, the abutment and crown fabrication processes were initiated. then the patients with the inclusion criteria were assigned to two groups to receive either stock or customized abutments.

Of all, 28 dental implants received stock abutments and 36 implants received customized abutments. Also, 28 dental implants were at the premolar site and 36 were at the molar site. Impressions were made with the conventional technique using addition silicone impression material (Betasil Vario Light, Muller-Omicron, Germany). In patients who required customized abutments , after pouring the impression with type IV dental stone (Vel-Mix Stone, Kerr) Scan bodies (DIO) were tightened on the cast, and the casts were placed in a scanner (DOF, Seoul, Korea). The abutments were designed in Exocad software following Exocad guidelines (Exocad GmbH, Darmstadt, Germany). the margin of the abutments was placed up to 1 mm subgingival and then the design was imported to Hyperdent software (FOLLOW-ME! Technology Group, Munich, Germany) for subsequent transfer to the milling machine. The customized abutment) ARUM, Doowon, Korea (was milled in a titanium milling machine (ARUM 5X-200, Doowon, Korea), and the procedure of the cement-retained and screw-retained crown fabrication and preparation was performed according to the standard conventional technique.

Using Aluminum oxide polishing cups, the sequential polishing of the abutment was performed to achieve optimal results in polishing customized abutments, promoting better integration with gingival tissues and overall implant success. First a coarser polishing instrument was used and then gradually progress to finer instruments were used to enhance the surface smoothness [10]. The abutment and the crown were delivered to the dental clinician. In the clinic, the abutment was tightened on the fixture, and the rest of the procedure for crown delivery was performed following the standard guidelines.

For patients who required a stock abutment, the abutment was ordered to the company with the desired angulation and gingival height, and then tightened on the fixture. Other steps of cement-retained crown delivery were performed conventionally.

Totally, 57 implants received full ceramic crown as prosthetic treatment and 7 implants received PFM crown.

All patients underwent clinical periodontal examination on the day of crown delivery and their pocket probing depth (PPD), papilla bleeding index (PBI) [11], keratinized width (KGW), plaque index (PI) [12] and modified gingival index (MGI) [13] were measured around every dental implant by a dental clinician, and recorded.

On the same day, a periapical radiograph was obtained from the implant using XCP intraoral positioner. The patients were recalled 12 months after prosthetic crown delivery. On the follow-up session, all periodontal parameters were measured by the same dental clinician again, and a periapical radiograph was obtained from the implant using XCP intraoral positioner and the same X-ray machine.

The two radiographs were compared to calculate the MBL. For this purpose, the contact line of the abutment with the upper border of the fixture on the radiograph at the mesial and distal of dental implant was selected as the reference. The first point of bone visualization at the mesial and distal of implant was marked, and the distance between each point to the reference point of the same side was measured and recorded as the MBL of the respective side. The mean of the two values was then calculated and multiplied by the actual implant height divided by the radiographic implant height (to include radiographic distortion) and recorded as the MBL around implant [14]. The survival rate and success rate of dental implants were calculated using implant success criteria introduced by Buser et al, [16]; No permanent radiographic translucency around dental implants, no sign of infection or puss discharge, no permanent pain, no dysesthesia, no foreign body reaction, grade 0-1 mobility.

Data distribution was analyzed by the Shapiro-Wilk test, which revealed non-normal distribution of data. Thus, the non-parametric Mann-Whitney U test was used to compare the stock and customized abutments and molar and premolar areas regarding MBL and periodontal parameters. The dplyr, rstatix, and PMCMR plus packages of R2 software version 4.2.1 were used for statistical analyses using the Mann-Whitney U test.

## Results

This cohort study was conducted on 64 patients (32 male and 32 female).26 implants were inserted in the maxilla and 38 implants were placed in the mandible. The study population was consisted of 28 stock abutments and 36 customized abutments.

57 implants received full ceramic crown as prosthetic treatment and 7 implants received PFM crown. Table-1 presents the frequency distribution of patients’ sex, type of jaw (maxilla/mandible), quadrant (right/left), crown material, and crown type in the two study groups.

The mean PBI of the two groups of stock and customized abutment were respectively, 0.42 and 0.03 in the baseline session, 0.69 and 0.62 in the follow-up session and there was no significant difference between them (P = 0.214).

The mean PPD of the two groups of stock and customized abutment were respectively, 1.52 mm and 1.90 mm in the baseline session, 1.78 mm and 1.65 mm in the follow-up session no significant difference was found between them (P = 0.051).

The mean MBL changed from 1.09 mm to 0.89 mm in stock abutments and from 0.78 mm to 0.84 mm in customized abutments during 1 year. There was no significant difference between the two abutments regarding this parameter (P = 0.095).

PI, MGI and KGW were the other three parameters that did not differ significantly (P˃0.05) between the two sessions (Table-2).

The mean bone level change between the two sessions in molars region was 0.08mm and in premolars region was 0.01mm. The two areas (P = 0.106) were not significantly different regarding MBL (Table-3).

## Discussion

**Table:** Table1. Frequency distribution of patients’ sex, type of jaw (maxilla/mandible), quadrant (right/left), crown material, and crown type in the two abutment groups

**Variable**	**Abutment**	**Category**	**Number**	**Percentage**
	Stock	Male	10	36 **٪**
**Sex**		Female	18	64 **٪**
	Customized	Male	22	61 **٪**
		Female	14	39 **٪**
	Stock	Maxilla	12	43 **٪**
**Jaw**		Mandible	16	57 **٪**
	Customized	Maxilla	14	39 **٪**
		Mandible	22	61 **٪**
	Stock	Right	14	50 **٪**
**Quadrant**		Left	14	50 **٪**
	Customized	Right	15	42 **٪**
		Left	21	58 **٪**
	Stock	PFM	0	0 **٪**
**Crown material**		Full ceramic	28	100 **٪**
	Customized	PFM	7	20 **٪**
		Full ceramic	29	80 **٪**
	Stock	Cement-retained	28	100 **٪**
**Crown type**		Screw-retained	0	0 **٪**
	Customized	Cement-retained	30	83 **٪**
		Screw-retained	6	17 **٪**

**Table:** Table2. Comparison of stock and customized abutments regarding MBL and periodontal parameters

**Variable**	**Abutment type **	**Time**	**Mean**	**Std. deviation **	**P-value**
	Stock	Baseline	0.42	0.77	
**Papilla bleeding index** (0-4 score)		Follow-up session	0.69	0.59	0.214
	Customized	Baseline	0.03	0.09	
		Follow-up session	0.62	0.51	
	Stock	Baseline	1.52	0.81	
**Pocket probing depth** (mm)		Follow-up session	1.78	0.67	0.051
	Customized	Baseline	1.90	1.25	
		Follow-up session	1.65	0.77	
	Stock	Baseline	0	0	
**Plaque index** (0-1 score)		Follow-up session	0.66	1.01	0.318
	Customized	Baseline	0.08	0.32	
		Follow-up session	0.46	0.97	
	Stock	Baseline	0.25	0.44	
**Modified gingival index** (0-4 score)		Follow-up session	0.43	0.87	0.51
	Customized	Baseline	0.06	0.23	
		Follow-up session	0.25	0.44	
	Stock	Baseline	2.78	2.50	
**Keratinized gingiva width** (mm)		Follow-up session	2.75	2.38	0.246
	Customized	Baseline	3.07	2.44	
		Follow-up session	2.61	2.10	
	Stock	Baseline	1.09	0.78	
**Marginal bone loss** (mm)		Follow-up session	0.89	0.61	0.095
	Customized	Baseline	0.78	0.57	
		Follow-up session	0.84	0.71	

**Table:** Table3. Comparison of MBL around dental implants in molar and premolar regions

**Variable**	**Region**	**Time**	**Mean** **(mm)**	**Std. deviation** **(mm)**	**P-value**
	Molar	Baseline	0.84	0.54	
**Marginal bone loss** (mm)		Follow up	0.76	0.70	0.106
	Premolar	Baseline	1.00	0.84	
		Follow up	0.99	0.61	

Studies comparing stock and customized titanium abutments are not many [7][17]. Thus, this study compared MBL and other periodontal parameters between stock and customized titanium abutment groups after a 1-year follow-up. The two groups of abutments had no significant difference regarding gender distribution, jaw type (maxilla/mandible), or quadrant (right/left) of implants; although differences in these parameters would have no significant effect on the results as these parameters were not the same in the test and control groups in some previous studies either [7][9]. Romeo et al. [18] reported similar success rate and survival rate of implants inserted in the maxilla and mandible. In the present study, the survival rate was 100% in both abutment groups, indicating that none of the implants had any problem during the one-year study period. Such a high survival rate was comparable to another study that compared stock and customized abutments [19].

The success rate was 100% in both abutment groups according to the criteria suggested by Buser et al, [16] which was close to the 89% success rate for stock abutments and 90% success rate for customized abutments reported in a previous study [19]. Small differences in values may be due to the fact that the abovementioned study was conducted on stock and customized zirconia abutments in the anterior region, and the problems involved crown fracture and patient dissatisfaction with the crown appearance [19].

In the present study, PI, PBI, and MGI were almost zero in the first session but they increased over time as measured in the follow-up session. However, the increase was not statistically significant in any parameter, and all peri-implant tissues in both groups had optimal health status. These results were in agreement with the previous findings [7][9][19]. Since the abutments are not exposed to the oral environment, PI appears to be mainly related to the oral hygiene status of patients rather than the abutment type [20]. Thus, increased PI may be due to higher motivation of patients for oral hygiene practice within the first days after implant insertion compared to the time of follow-up. Also, patients often brush their teeth prior to visiting a dentist. Thus, the measured PI cannot be a true representative of the actual PI of patients. The trend of change in PPD over one year had no significant difference between the two groups, which was consistent with previous findings [7][9][19][21].

Peri-implant MBL is among the most important criteria for prediction of implant success [22][23]. In the present study, the mean MBL was -0.20 mm in the stock abutment group, and +0.06 mm in the customized abutment group. All MBL values indicated presence of sufficient volume of bone around dental implants in both groups since the MBL did not exceed 1.5 mm in any group, which was in agreement with previous findings [21].

Assessment of MBL revealed an increase in crestal bone level in some cases, which has also been reported in some other studies [7][21][24]. Thus, as recommended by Schpke et al, the term "bone level changes" may be preferred to "bone loss" in such cases [7]. Borzongy et al. [20] assessed the bone level in the first prosthetic treatment session, at the session of crown delivery, and at the 1- and 6-month follow-ups. They noticed slight apical tilting of bone level at the 1- and 6-month follow-ups; however, the bone level had a slight coronal tilt at the 1-year follow-up. Merli et al. [25] suggested the use of radiographic MBL index and BOP for clinical diagnosis of peri-implant disease. In the present study, the two abutment groups had no significant difference in buccal KGW, which was in line with the results of Schpke et al [7]. However, a multi-center clinical trial compared stock and customized CAD/CAM titanium and zirconia abutments regarding labial KGW and gingival recession after 2 years. They reported superior performance of CAD/CAM titanium abutments compared to others; however, since zirconia abutments had been selected for cases with up to 2 mm of labial KGW, and titanium abutments had been selected for cases with > 2 mm of labial KGW, risk of errors exists in their study [7].

Although some studies did not support the statement that absence of keratinized mucosa can compromise peri-implant soft tissue health [26][27], the results of a meta-analysis done by Lin et al, which was mainly conducted on cross-sectional studies suggested that presence of at least 1-2 mm of keratinized mucosa may be useful for reduction of plaque accumulation, inflammation, mucosal recession, and clinical attachment loss [28]. In the present study, all dental implants had optimal health after 1 year; therefore, amount of keratinized gingiva had no significant effect on the one-year treatment success.

## Conclusion

In the present study, the two abutment types had no significant difference in any clinical parameter. Thus, stock or CAD/CAM customized abutments may be selected according to secondary parameters such as software availability, personal preferences, easy fabrication, and cost. Therefore, both stock and customized titanium abutments can serve as valuable options for implant restoration. Future studies with a larger sample size and longer follow-up periods are recommended.

## Conflict of Interest

None.
